# Antiproliferative Effect of 7-Ketositosterol in Breast and Liver Cancer Cells: Possible Impact on Ceramide, Extracellular Signal-Regulated Kinases, and Nuclear Factor Kappa B Signaling Pathways

**DOI:** 10.3390/ph17070860

**Published:** 2024-07-01

**Authors:** Zerrin Barut, Mutay Aslan, Bürke Çırçırlı, Tuğçe Çeker, Çağatay Yılmaz

**Affiliations:** 1 Faculty of Dentistry, Antalya Bilim University, 07190 Antalya, Turkey; zerrin.barut@antalya.edu.tr; 2 Department of Medical Biochemistry, Faculty of Medicine, Akdeniz University, 07070 Antalya, Turkey; tugceker159@gmail.com (T.Ç.); ccagatayyilmaz@gmail.com (Ç.Y.); 3 Department of Medical Biotechnology, Institute of Health Sciences, Akdeniz University, 07070 Antalya, Turkey; burkecircirli@outlook.com

**Keywords:** 7-Ketositosterol, ceramide, apoptosis, ERK

## Abstract

**Background**: This study aimed to examine the effect of 7-Ketositosterol (7-KSS), on sphingomyelin/ceramide metabolites and apoptosis in human breast MCF-7 and human liver HepG2 cancer cells. **Methods**: Anti-proliferative effects of 7-KSS treatment were assessed at different concentrations and periods. Cell viability was assessed through MTT analysis, whereas the levels of sphingosine-1-phosphate (S1P), sphingomyelins (SMs), and ceramides (CERs) were measured using LC-MS/MS. Phosphorylated 44/42 ERK1/2 and NF-κB p65 (Ser536) protein levels were measured by Western blot analysis and immunofluorescence staining. Apoptosis was evaluated by TUNEL staining and flow cytometric assessment of annexin-V and propidium iodide (PI) labeling. **Results**: Treatment with 7-KSS significantly decreased cell survival and S1P, p-44/42 ERK1/2, and p-NF-κB p65 protein levels in cancer cells compared to controls. A substantial rise was detected in intracellular amounts of C16-C24 CERs and apoptosis in cancer cells incubated with 7-KSS. **Conclusions**: 7-KSS stimulated ceramide accumulation and apoptosis while decreasing cell proliferation via downregulating S1P, p-44/42 ERK1/2, and p-NF-κB p65 protein levels.

## 1. Introduction

The protective effects of dietary plant sterols in different cancers have been reported by recent studies [1,2]. 7-Ketositosterol is a natural substance derived from oxidized plant sterols [3], also named phytosterols. Phytosterols resemble cholesterol and are generally known for their ability to help lower cholesterol levels in humans by blocking the absorption of cholesterol in the intestines [4]. Common phytosterols include beta-sitosterol which is one of the most abundant phytosterols found in plants such as nuts, seeds, fruits, and vegetables. Beta-sitosterol is known for its cholesterol-lowering effects and is often used as a dietary supplement for managing cholesterol levels [5]. Campesterol is another common phytosterol found in various plant oils, seeds, and nuts. Like beta-sitosterol, campesterol also has cholesterol-lowering properties [6]. Stigmasterol is found in soybeans, vegetables, and various grains. Stigmasterol is structurally like cholesterol and has been studied for its potential role in reducing cholesterol levels and inflammation [6]. Brassicasterol is predominantly found in brassica vegetables like broccoli, brussels sprouts, and cabbage. Brassicasterol has also been studied for its cholesterol-lowering effects. There is some evidence to suggest that phytosterols may modulate immune function, although more research is needed in this area [6].

Some studies suggest that phytosterols may possess anti-inflammatory properties, which could be beneficial for conditions associated with inflammation, such as arthritis and cardiovascular diseases [7]. Phytosterols may act as antioxidants, helping to neutralize harmful free radicals in the body and reduce oxidative stress, which are implicated in various diseases and aging processes [7]. In the context of cancer, phytosterols have attracted some interest because of their structural similarity to cholesterol and potential to reduce the risk of certain cancers through various mechanisms, such as reducing inflammation, modulating immune responses, or interfering with cancer cell growth pathways [8]. Beta-sitosterol’s anticancer actions have been linked to promoting apoptosis, triggering cell cycle arrest, controlling oxidative stress, increasing metabolic reprogramming, preventing invasion and metastasis, adjusting immunology and inflammation, and thwarting drug resistance [9]. Regarding apoptosis, β-sitosterol acts via several different pathways. For example, β-sitosterol regulates p53 to cause apoptosis in A549 lung cancer cells via altering components like p21 and p53 [10]. Apoptosis in gastric cancer is triggered by interactions with Bcl-2, pro-caspase-3, and Bax [10]. Likewise, death receptors and Bcl-2 are involved in both intrinsic and extrinsic apoptotic pathways that are activated in HepG2 cells [10]. β-sitosterol also acts on cell cycle arrest through several different mechanisms. For example, β-sitosterol induces cell cycle arrest in lung adenocarcinoma by blocking Cyclin-D1 and cyclin-dependent kinase 2 (CDK-2) [10]. Regarding cancer migration, β-sitosterol mainly acts through the protein kinase B/glycogen synthase kinase 3 beta (AKT/GSK-3b) and epithelial–mesenchymal transition (EMT) signaling pathways [10].

Phytosterols contain double bonds in their chemical structure, making them susceptible to oxidation [11]. Free radicals can abstract hydrogen atoms from the double bonds, leading to the formation of phytosterol oxidation products (POPs) [11]. Transition metal ions such as iron and copper can catalyze the oxidation of phytosterols by facilitating the generation of reactive oxygen species. Exposure to light, particularly ultraviolet (UV) radiation, can promote the oxidation of phytosterols [12]. The oxidation of phytosterols can result in the formation of various oxidation products, including hydroperoxides, epoxides, aldehydes, and ketones [11]. These oxidized phytosterols may have altered chemical properties and reduced biological activity compared to their unoxidized counterparts [13]. Moreover, some oxidation products may have cytotoxic effects [14,15]. 7-Ketositosterol itself is a specific oxidation product of beta-sitosterol. It is reported to be the most abundant POP in commercial spreads [16]. Previous studies have shown that 7-KSS is cytotoxic and apoptotic in HepG2 human liver cancer cells [14]. Phytosterols boost ceramide levels in human prostate, colon, and breast cancer cells [17]. The increase in ceramide levels blocks cell proliferation, activates caspases, and leads to apoptosis in cancer cells [18].

Extracellular signal-regulated kinases (ERKs), also known as mitogen-activated protein kinases (MAPKs), show an important function in cancer pathogenesis [19]. Phytosterols have been clearly recognized in the regulation of ERK signaling [20]. It is also known that ceramide inhibits ERKs consisting of ERK1 and ERK2 [21]. The ERK proteins receive an input from the upstream toll-like receptor 4 (TLR4), which stimulates the activated protein 1 (AP1) signaling pathway, leading to the expression of genes and inflammatory mediators related to cell cycle regulation [22]. Nuclear factor kappa B (NF-κB) and ERKs are described as adjusting the expression of the cell cycle genes, causing an increase in cell growth, proliferation, and angiogenesis [23]. Numerous phytosterols inhibit tumor growth and diminish the tumor microenvironment by preventing the activation of NF-κB and ERK signaling, as well as the production of cytokines [20].

The biological significance of 7-KSS is still an area of research, and it is not as well-known or understood as other related compounds. In this study, we investigated the anti-proliferative and anticancer efficacy of 7-KSS in human breast (MCF-7) and liver (HepG2) cancer cells by measuring intracellular sphingolipid metabolite levels and apoptosis. Studies have reported that TLR-4 is expressed in both cancer cell lines [24,25]. Thus, we also determined the effects of 7-KSS on downstream effectors of the ERK signaling pathway by determining phospho-NF-κB and phospho-ERK protein levels. We report herein that 7-KSS can increase ceramide levels, induce apoptosis, and inhibit cell proliferation by blocking the phosphorylation of ERKs and NF-κB in human liver and breast cancer cells.

## 2. Results

### 2.1. Cell Viability Analysis

**Figure 1:**
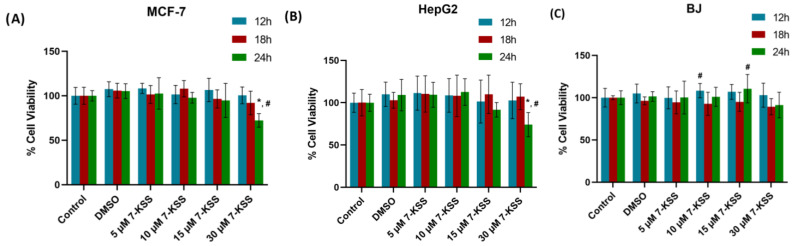
Analysis of 7-Ketositosterol toxicity in cancer cells and BJ fibroblasts. (**A**) 24 h viability analysis of 7-KSS administration in MCF-7 cells. Values mean ± SD (*n* = 8). One-way ANOVA or Kruskal–Wallis analysis was performed for statistical testing. The difference between the groups was analyzed by Tukey or Dunn’s test. *, *p* < 0.05 vs. control, DMSO, and 5 μM 7-KSS groups over the same time periods. #, *p* < 0.05 compared to 12 h and 18 h in the same dose interval. (**B**) 24 h viability analysis of 7-KSS administration in HepG2 cells. Values mean ± SD (*n* = 8). One-way ANOVA or Kruskal–Wallis analysis was performed for statistical testing. The difference between the groups was analyzed by Tukey or Dunn’s test. *, *p* < 0.05 vs. control, DMSO, 5 μM 7-KSS, and 10 μM 7-KSS over the same time periods. #, *p* < 0.05 compared to 12 and 18 h in the same dose range. (**C**) 24 h viability analysis of 7-KSS administration in BJ fibroblast cells. Values mean ± SD (*n* = 7–8). One-way ANOVA or Kruskal–Wallis analysis was performed for statistical testing. The difference among the groups was analyzed by Tukey or Dunn’s test. #, *p* < 0.05 vs. 18 h at the same dose.

Viability analysis following 24 h 7-KSS treatment was performed in cancer cells and non-cancerous BJ fibroblasts (Figure 1). Treatment of MCF-7 cells with 5, 10, or 15 μM 7-KSS did not significantly affect cell viability over time. It was observed that 30 μM 7-KSS application significantly reduced cell viability in breast cancer cells by approximately 30% in 24 h (Figure 1A). Incubation of liver cancer cells with 5–30 μM 7-KSS for 12–18 h did not significantly affect cell viability compared to control and DMSO groups (Figure 1B). HepG2 cell viability was reduced significantly by approximately 25% after 24 h of 30 μM 7-KSS treatment. It was determined that 5–30 μM 7-KSS administration in the non-cancerous BJ fibroblast cell line did not significantly affect cell viability at 24 h (Figure 1C).

### 2.2. Proliferating Cell Nuclear Antigen Levels

**Figure 2:**
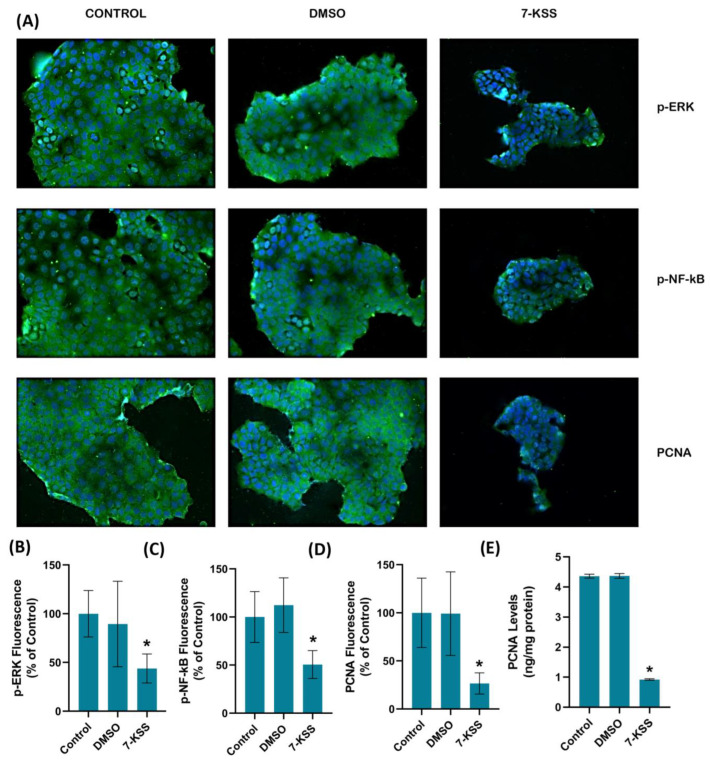
(**A**) Representative Immunofluorescent staining of p-ERKs, p-NF-κB, and PCNA in MCF-7 cells incubated with 30 µM 7-Ketositosterol for 24 h. A 40× objective lens was used to obtain double-labeled images. (**B**) Quantitation of p-ERK fluorescence staining by ImageJ software. Values mean ± SD (*n* = 10). Kruskal–Wallis test and Dunn’s multiple comparison analysis were performed to test statistical significance. * *p* < 0.05 vs. control and DMSO groups. (**C**) Quantitation of p-NF-κB fluorescence staining. Values are given as mean ± SD (*n* = 10). One-way ANOVA test and Tukey’s multiple comparisons were used to determine statistical significance. * *p* < 0.05 compared to control and DMSO. (**D**) PCNA staining quantitation. Values are given as mean ± SD (*n* = 10). One-way ANOVA test and Tukey’s multiple comparisons were used to determine statistical significance. * *p* < 0.05 compared to control and DMSO. (**E**) PCNA levels in MCF-7 cells. Values mean ± SD (*n* = 5). *, *p* < 0.05 vs. control and DMSO groups.

**Figure 3:**
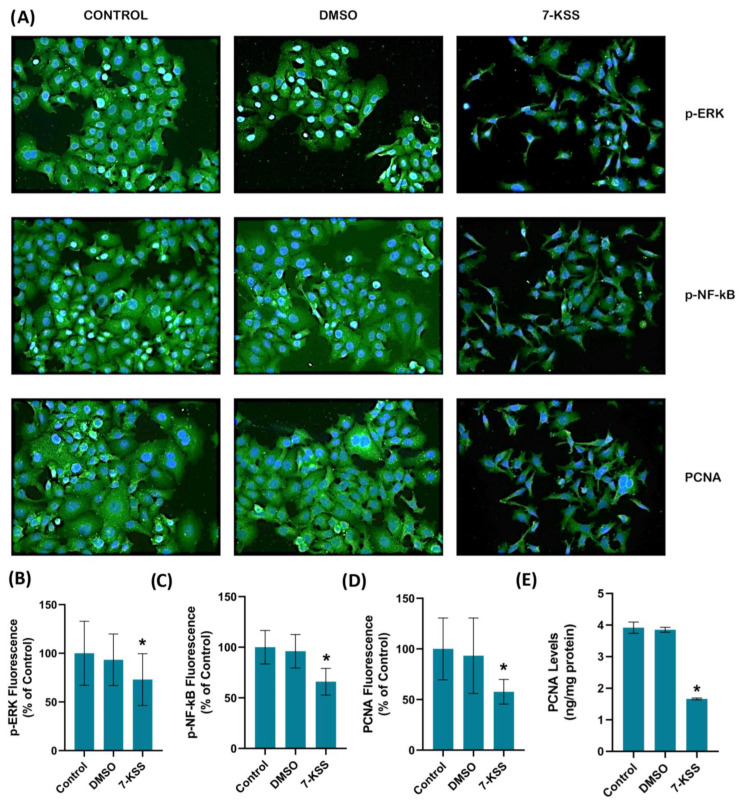
(**A**) Representative immunofluorescent staining of p-ERKs, p-NF-κB, and PCNA in HepG2 cells incubated with 30 µM 7-Ketositosterol for 24 h. A 40× objective lens was used to obtain double-labeled images. (**B**) Quantitation of p-ERK fluorescence staining by ImageJ software. Values mean ± SD (*n* = 10). Kruskal–Wallis test and Dunn’s multiple comparison analysis were performed to test statistical significance. * *p* < 0.05 vs. control group. (**C**) Quantitation of p-NF-κB fluorescence staining. Values are given as mean ± SD (*n* = 10). One-way ANOVA test and Tukey’s multiple comparisons analysis were used for statistical analysis. * *p* < 0.05 compared to control and DMSO groups. (**D**) Quantitation of PCNA fluorescence staining by ImageJ software. Values mean ± SD (*n* = 10). Kruskal–Wallis test and Dunn’s multiple comparison analysis were performed to test statistical significance. * *p* < 0.05 vs. control and DMSO groups. (**E**) PCNA levels in HepG2 cell groups. Values mean ± SD (*n* = 5). One-way ANOVA and Tukey multiple comparison test were performed for statistical analysis. *, *p* < 0.05 vs. control and DMSO groups.

Figure 2A and Figure 3A show PCNA immunofluorescence staining in MCF-7 and HepG2 cells incubated with 30 µM 7-KSS for 24 h. Quantitation of PCNA fluorescence staining showed that PCNA protein was significantly suppressed in MCF-7 cells (Figure 2D) and HepG2 cells (Figure 3D) treated with 7-KSS compared to control and DMSO groups. ELISA analysis of PCNA protein levels in MCF-7 (Figure 2E) and HepG2 (Figure 3E) cells confirmed immunofluorescence staining and showed that the amount of PCNA protein was meaningfully reduced in cancer cells incubated with 30 µM 7-KSS for 24 h vs. control and DMSO groups.

### 2.3. Sphingolipid Levels

Sphingolipid levels measured in cancer cells are shown in Table 1. A meaningful rise was detected in endogenous C18-C24 CERs levels in MCF-7 and HepG2 cells treated with 30 µM 7-KSS for 24 h, compared to control and DMSO groups. Incubation with 30 µM 7-KSS for 24 h considerably reduced S1P levels in cancer cells.

### 2.4. Phospho-ERK and Phospho-NF-κB p65 Protein Levels

**Figure 4:**
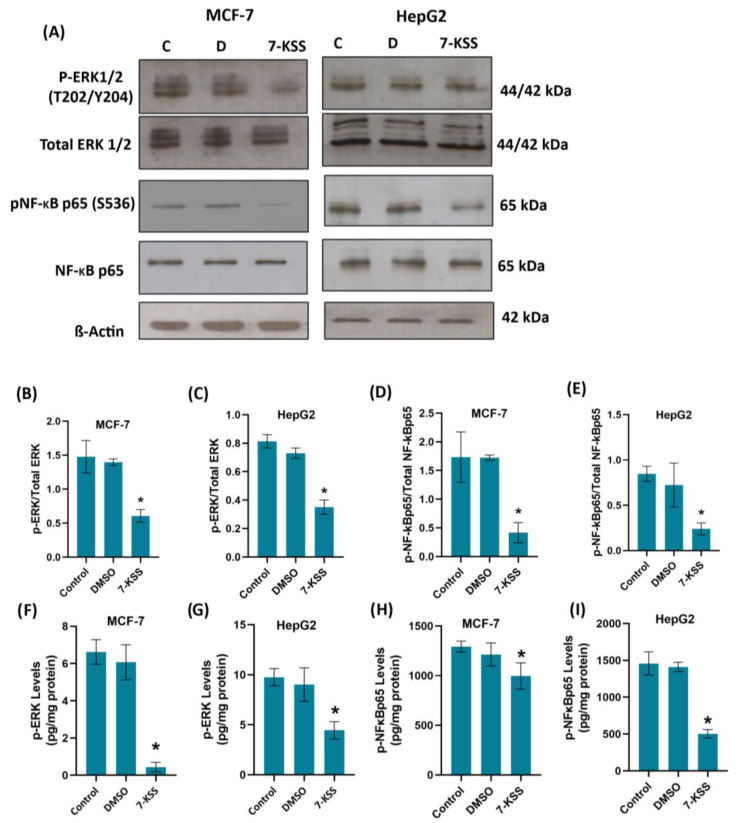
(**A**) Illustrative Western immunoblots for phospho-44/42 ERK1/2 (T202/Y204), total ERK 1/2, phospho-NF-κB p65 (S536), total NF-κB p65, and β-Actin in MCF-7 and HepG2 cells. C, control; D, cells treated with 1 μL/mL dimethyl sulfoxide; 7-KSS, cell treated with 30 μM 7-KSS for 24 h. (**B**) The band quantitation ratio of phospho-ERK/Total ERK in MCF-7 and (**C**) HepG2 cells. (**D**) The band quantitation ratio of phospho-NF-κB p65/Total NF-κB p65 in MCF-7 and (**E**) HepG2 cells. Blots were quantitated by ImageJ software. Data shown are representative of 3 separate experiments and values are given as mean ± SD. Statistical analysis was performed by one-way ANOVA and all pairwise multiple comparison procedures carried out by the Tukey test. *, *p* < 0.01 vs. control and DMSO groups. (**F**) Phospho-ERK levels in MCF-7 cells. Values mean ± SD (*n* = 5–8). One-way ANOVA and Tukey multiple comparisons were used to determine statistical significance. *, *p* < 0.05 vs. control and DMSO groups. (**G**) Phospho-ERK levels in HepG2 cells. Values mean ± SD (*n* = 8). One-way ANOVA and Tukey multiple comparisons were performed for statistical analysis. *, *p* < 0.05 when compared with the control and DMSO groups. (**H**) Phospho-NF-κB p65 levels in MCF-7 cells. Values mean ± SD (*n* = 8). One-way ANOVA and Tukey multiple comparisons were performed to test statistical significance. *, *p* < 0.05 when compared with the control and DMSO groups. (**I**) Phospho-NF-κB p65 levels in HepG2 cells. Values mean ± SD (*n* = 8). One-way ANOVA and Tukey multiple comparisons were used to determine statistical significance. *, *p* < 0.05 when compared with the control and DMSO groups.

Figure 2A and Figure 3A show immunofluorescent staining of p-ERKs and p-NF-κB in MCF-7 and HepG2 cells treated with 30 µM 7-KSS for 24 h. Quantitation of p-ERK and p-NF-κB fluorescence staining showed that the phosphorylated proteins were significantly suppressed in MCF-7 cells (Figure 2B,C) and HepG2 cells (Figure 3B,C) treated with 7-KSS compared to control and DMSO groups. Western blot analysis also confirmed immunofluorescence staining of p-ERKs and p-NF-κB in cancer cells (Figure 4A). Significantly decreased p-ERK/Total ERK and p-NF-κB p65/Total NF-κB p65 ratios in both cancer cells agreed with the immunofluorescence data (Figure 4B–E). ELISA analysis of p-ERK and p-NF-κB protein levels in MCF-7 (Figure 4F,H) and HepG2 (Figure 4G,I) cells confirmed the immunofluorescence and Western blot analysis, and showed that phosphorylated proteins were significantly reduced in cancer cells incubated with 30 µM 7-KSS for 24 h compared to the control and DMSO groups.

### 2.5. Apoptosis

**Figure 5:**
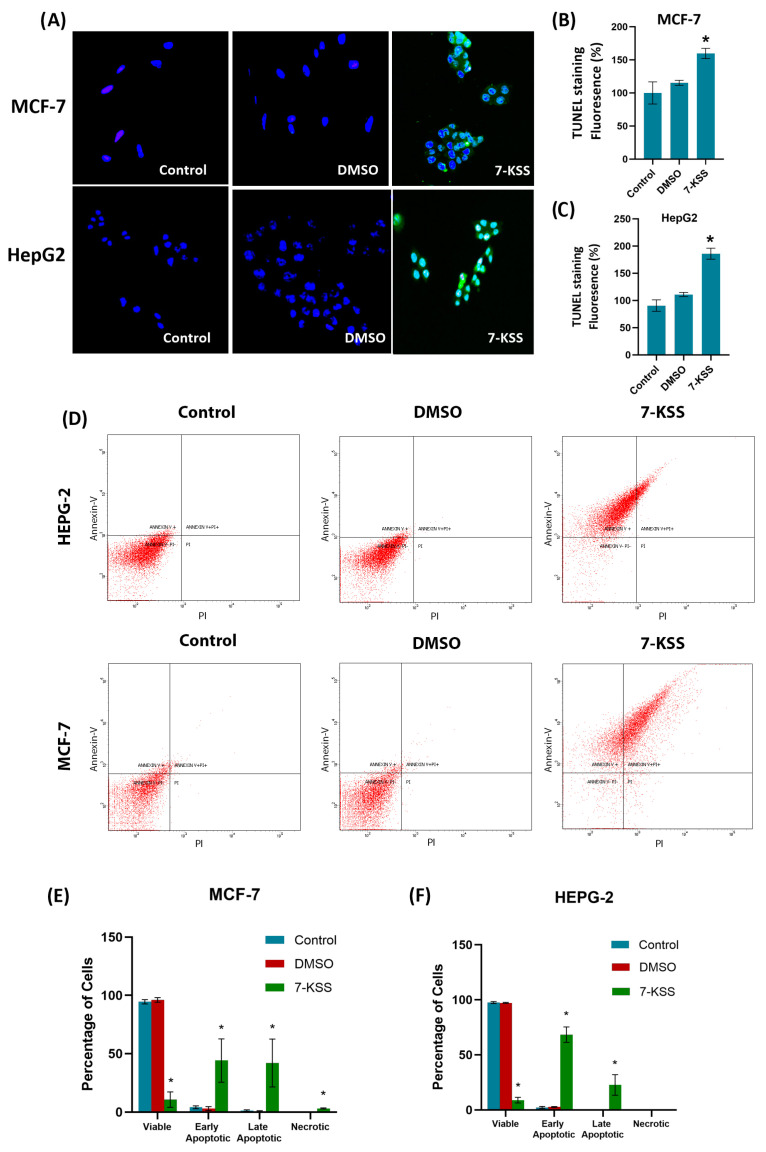
TUNEL staining in cancer cells. (**A**) Dimethyl sulfoxide (DMSO, 1 μL/mL) and 7-KSS (30 μM) were applied for 24 h (200× magnification). (**B**) Quantitation of TUNEL staining in MCF-7 cells with the ImageJ program. Values mean ± SD (*n* = 7–8). Kruskal–Wallis test and Dunn’s analysis were performed to test statistical significance. *, *p* < 0.05 compared with the control and DMSO groups. (**C**) Quantitation of TUNEL staining in HepG2 cells with ImageJ program. Values are mean ± SD (*n* = 8). One-way ANOVA and Tukey multiple comparisons were used to determine statistical significance. *, *p* < 0.05 when compared with the control and DMSO groups. (**D**) Annexin V–FITC and PI-labeled HepG2 and MCF-7 cells. In total, 10,000 events were analyzed for each condition. (**E**) Quantitative analysis of annexin-V and PI labeling in MCF-7 cells by flow cytometry. Values mean ± SD (*n* = 3). One-way ANOVA analysis and Tukey test were used for statistical analysis. *, *p* < 0.05 compared to control and DMSO groups. (**F**) Quantitative analysis of annexin-V and PI labeling in HepG2 cells by flow cytometry. Values mean ± SD (*n* = 3). One-way ANOVA analysis and Tukey test were used for statistical analysis. *, *p* < 0.05 compared to control and DMSO groups.

TUNEL analysis showed a significant increase in apoptosis in cancer cells treated with 30 µM 7-KSS for 24 h (Figure 5A). Quantitation of apoptosis via fluorescence staining showed a significant increase in apoptotic MCF-7 (Figure 5B) and HepG2 (Figure 5C) cells treated with 7-KSS compared to the control and DMSO groups. Figure 5D displays illustrative plots from HepG2 and MCF-7 cells. In individual squares, the left inferior corner displays living cells (annexin V negative/PI negative), the left higher corner displays early apoptotic cells (annexin V positive), the right higher corner indicates necrotic-late apoptotic cells (annexin V positive/PI positive), and the right inferior corner (PI positive) characterizes necrotic cells. Annexin V is a protein that has a high affinity for phosphatidylserine (PS), a phospholipid normally found on the inner leaflet of the plasma membrane. During apoptosis, PS is translocated from the inner to the outer leaflet of the plasma membrane, exposing it to the extracellular environment. Annexin V can bind specifically to PS. PI is a fluorescent dye that attaches to DNA but is unable to penetrate live cells possessing intact membranes. Nonetheless, it can infiltrate cells with compromised membranes, like those in late apoptosis or necrosis. Consequently, employing both Annexin V and PI staining allows differentiation between live cells, early apoptotic, late apoptotic, and necrotic cells. The flow cytometric quantification of annexin V–FITC labelling in MCF-7 and HepG2 cells is presented in Figure 5E and Figure 5F, respectively. Treatment with 7-KSS significantly increased the number of early and late apoptotic cells compared to the control.

## 3. Discussion

The findings of the current investigation revealed that treatment of MCF-7 and HepG2 cancer cells with 5–15 μM 7-KSS did not significantly alter cell viability over a 24 h period. Cancer cell viability was reduced significantly after 24 h 30 μM 7-KSS treatment. Our result is in accord with a study showing that the critical 7-KSS concentration to accomplish subtoxic injury in HepG2 cells at 24 h was 30 μM [8]. Another study treated U937 human lung lymphoblastic cells with beta-sitosterol oxides and reported that after 12 h, the survival of cells incubated with 120 µM beta-sitosterol oxides was decreased by approximately 50% compared to the control [26]. Providentially, in the case of noncancerous cells, 7-KSS caused no toxic effect even at 30 μM, signifying negligeable cytotoxicity for normal tissues.

A meaningful rise was detected in intracellular quantities of C18–C24 ceramides in HepG2 and MCF-7 cells incubated with cytotoxic amounts of 7-KSS for 24 h, relative to controls. This is the first study evaluating the effect of 7-KSS on endogenous sphingolipid levels in breast and liver cancer cells. Our findings agree with an earlier report indicating that beta-sitosterol action boosted ceramide amounts in HT-29 colon cancer cells [27]. Likewise, 16 µM beta-sitosterol was administered to LNCaP human prostate cancer cells and it was reported to cause apoptosis, which was in adjunct with a significant increase in ceramide production [28]. Investigators also studied the outcome of nutritional β-sitosterol on cell growth and ceramide levels in MCF-7 and MDA-MB-231 human breast cancer cells. The findings showed that beta-sitosterol activated de novo synthesis of ceramide by increasing serine palmitoyl transferase activity [29]. We also detected that treatment with 30 µM 7-KSS for 24 h considerably reduced S1P levels in cancer cells. To the best of our knowledge, this study is the first to report reduced S1P levels in cancer cells treated with 7-KSS. Functional sphingolipid metabolites such as ceramide and S1P have vital functions in the action of numerous biological pathways that are central to cancer pathogenesis. Current knowledge on biologically active sphingolipid synthesis recognizes their vital roles in both cancer development and advancement. Ceramide, a key component in sphingolipid metabolism, acts as a tumor-suppressing molecule, triggering anti-proliferative and apoptotic reactions in numerous cancerous cells [30]. On the contrary, S1P brings on reactions that make it a cancer-supporting lipid.

Our findings do not provide a direct explanation for how 7-KSS promotes ceramide formation in HepG2 and MCF-7 cells. One mechanism might be that certain ceramide synthesis pathway enzymes are susceptible to changes in membrane fluidity brought on by the addition of phytosterols with a structure distinct from cholesterol. A prior study observed a reduction in the fluidity of hepatic membranes in rats fed a diet consisting of 2% cholesterol and 3% β-sitosterol for a duration of 21 days [31]. For membrane-bound enzymes to function at their optimum, certain levels of membrane fluidity may be necessary [32]. Like beta-sitosterol, 7-KSS, an oxidation product of beta-sitosterol, may similarly cause an increase in the formation of ceramides by upregulating enzymes involved in ceramide biosynthesis such as serine palmitoyltransferase (SPT) and ceramide synthase (CerS) [29,33]. As with campesterol, 7-KSS could also downregulate ceramidases, which are enzymes that degrade ceramides into sphingosine and fatty acids, thereby increasing ceramide levels [34].

We have seen that phosphorylated 44/42 ERK1/2 and phosphorylated NF-κB p65 (Ser536) proteins were significantly suppressed in cancer cells treated with 7-KSS compared to the control groups. To the best of our knowledge, this is the first study reporting that 7-KSS down regulates phosphorylation of ERK1/2 and NF-κB in cancer cells. It is, however, known that 7-*alpha*-OH-*beta*-sitosterol, an uncommon biological phytosterol oxide, caused decreased cell viability in MCF-7 cells by means of apoptosis through down regulation of the ERK1/2 signaling pathway [35]. Fucosterol, a phytosterol commonly obtained from marine algae and numerous plant types, was also found to inhibit the expression of phosphorylated ERK1/2 with no evident consequences on the expression of ERK1/2 [36]. Phospho-44/42 ERK1/2, also known as phosphorylated extracellular signal-regulated kinases 1 and 2, are crucial components of the MAPK signaling pathway [37]. Aberrant phosphorylation of ERK1/2 points to pathogenesis of tumor development and growth; thus, ERK1/2 has been identified as a potential therapeutic target for cancer [37]. This pathway plays a fundamental role in regulating various cellular processes including cell proliferation, differentiation, survival, and apoptosis. ERK1/2 phosphorylation is closely associated with cell proliferation and growth [38]. Phosphorylated ERK1/2 signaling is also implicated in the process of cellular differentiation [38]. ERK1/2 phosphorylation can promote cell survival by inhibiting apoptosis in cancer cells [39]. 7-Ketositosterol may block upstream activators like Ras to stop ERK signaling [40] or increase the activity of MAPK phosphatases that deactivate ERKs [41]. As reported with beta-sitosterol, 7-KSS might also interact with membrane receptors or signaling molecules that negatively regulate ERK activation [42].

Different phytosterols have been described for their antitumor actions by preventing NF-κB activation and decreasing inflammatory cytokines and chemokines focusing on NF-κB signaling [20]. NF-κB p65, a transcription factor, possesses signals for nuclear localization, regions for binding to DNA, and two activation domains known as TA1 and TA2. The TA1 region, containing a C-terminal of 30 amino acids, serves as a pivotal transactivation domain. NF-κB transactivation relies on multiple phosphorylation events within this domain. One such crucial phosphorylation site, Ser536, located within TA1, remains conserved across various species. Phosphorylation of p65 enhances its transcriptional activity. [43]. Beta-sitosterol was also shown to downregulate phospho-NF-κB p65 levels, while having no influence on the total level of NF-κB p65 in pancreatic cancer cells [44]. 7-Ketositosterol might inhibit the activation of NF-κB by preventing the phosphorylation and degradation of IκBα (an inhibitor of NF-κB), leading to decreased transcription of pro-inflammatory genes [45]. 7-Ketositosterol may also have direct interactions with elements of the NF-κB signaling pathway, like blocking IκB kinase (IKK) activity, which would lower the activation of NF-κB [45].

As discussed, important mechanisms for the anticancer effects of phytosterols include stimulation of ceramide formation and induction of apoptosis. TUNEL analysis, as well as annexin V–FITC/PI staining, showed a significant increase in apoptosis in cancer cells incubated with 30 µM 7-KSS for 24 h. Our data support previous findings reporting that 7-KSS increased early apoptosis in human intestinal carcinoma cells [46], HepG2 cells [14], and human leukemic U937 cells [47].

Although basic cellular mechanisms and early drug testing can greatly benefit from cancer cell culture studies, it is important to recognize the limitations of this approach. In vivo models and clinical data should be added to these investigations to obtain a more thorough understanding of cancer biology and treatment responses. Cancer cell cultures do not replicate the complex tumor microenvironment present in living organisms, which includes interactions with stromal cells, immune cells, the extracellular matrix, and blood vessels. Conventional two-dimensional cell cultures are unable to replicate the three-dimensional architecture of tumors, which may have an impact on drug responsiveness and cell behavior. Cancer cell lines may differ from the original tumor and from one subculture to another because of genetic and phenotypic alterations throughout time. Continuous passage and adaptation to in vitro conditions can create selection pressures that favor the survival of specific cell populations, which may not represent the heterogeneity of the original tumor. The nutritional composition and oxygen concentrations in cell culture media can vary greatly from those in the tumor microenvironment, which may have an impact on drug responses and cellular metabolism. The pharmacokinetics and pharmacodynamics of drug delivery in cell cultures are not true representations of drug absorption, distribution, metabolism, and excretion in living organisms. Many cancer cell culture studies focus on a single type of cancer cell, which does not account for the heterogeneity within tumors that can affect treatment outcomes.

In summary, 7-KSS application significantly reduced cell viability, suppressed proliferating cell nuclear antigen, S1P, p-ERK, and p-NF-κB p65 protein levels while increasing ceramide amounts and apoptotic cell numbers in cancer cells. Increased ceramide concentrations, reduced S1P, decreased phospho-44/42 ERK1/2, and lowered phospho-NF-κB p65 levels contribute to decreased proliferation in cancer cells treated with 7-KSS, potentially facilitating apoptosis. Our findings suggest that 7-KSS has significant potential as a cancer therapeutic agent, primarily through its ability to induce apoptosis and modulate key signaling pathways. Future research should focus on translating these in vitro findings into clinical applications. Future studies using animal models of cancer could validate the in vitro findings reported herein. These studies would help us to understand the efficacy and safety of 7-KSS in a more complex biological system. The use of specific inhibitors or genetic models could also delineate the pathways involved in 7-KSS-induced apoptosis and the increase in ceramide levels. The consequences of 7-KSS treatment could also be evaluated in combination with other chemotherapy agents or targeted therapies to determine potential synergistic effects and enhance therapeutic efficacy. The use of patient-derived cancer cells and organoids could also better mimic the clinical scenario and validate the findings. Addressing these areas could advance the understanding of 7-KSS’s mechanisms of action and its potential role in cancer therapy, ultimately leading to more effective and targeted treatment options for patients.

## 4. Materials and Methods

### 4.1. Cell Culture

Human breast cancer (MCF-7), human liver cancer (HepG2), and human fibroblast (BJ) cell lines were obtained from American Type Culture Collection (ATCC; Manassas, VA, USA). MCF-7 cells were cultured using Dulbecco’s Modified Eagle’s Medium (DMEM)–F12 (Biowest; Cat.#L0092, Nuaillé, France) containing 10% (*v*/*v*) heat-inactivated fetal bovine serum (FBS) (Gibco, Life Technologies, Grand Island, NY, USA), 100 U/mL penicillin, 100 μg/mL streptomycin (Gibco), amphotericin B (5 μg/100 mL, Gibco), and 1% 2.5 mM L-Glutamine. HepG2 and BJ cells were cultured with DMEM high-glucose medium (Sigma; Cat.#D5648, St. Louis, MO, USA) containing 3.7 g/L sodium bicarbonate (Sigma-Aldrich), 10% (*v*/*v*) FBS, 100 U/mL penicillin, 100 μg/mL streptomycin, 1% sodium pyruvate (Gibco), and 5 μg/100 mL amphotericin B. The cells were propagated in the incubator at 37°C with 5% CO_2_ and 95% humidified air. When cells reached 80% confluency, they were passaged by removal from the flask surface using trypsin-EDTA (0.05% Trypsin/0.02% EDTA; Gibco) and suspended into new flasks.

### 4.2. 7-Ketositosterol Treatment

The 7-KSS used in our study was commercially available (MW = 428.7 g/mol; Cayman, Cat.#37189, Ann Arbor, MI, USA). An amount of 5 mg of 7-KSS was dissolved in 1 mL of DMSO to prepare an 11.66 mM stock solution. An intermediate stock of 1 mM was formed with cell culture medium. The final concentrations of 7-KSS used in MTT [3-(4,5-dimethylthiazol-2-yl)-2,5-diphenyltetrazolium bromide] analysis were 5, 10, 15, and 30 μM. MTT analysis data were used to determine the final dose and duration of 7-KSS administration in MCF-7 and HepG2 cells.

### 4.3. MTT Analysis

MCF-7, HepG2, and BJ cells were transferred to 96 well plates. Cells were incubated overnight to adhere, and later treated with culture medium containing 1 μL/mL DMSO or 5–30 μM 7-KSS. At the end of 12, 18, and 24 h incubation periods, MTT protocol was started. MTT (Gold Biotechnology Inc., St. Louis, MO, USA) was dissolved in PBS (5 mg/mL) and passed through a 0.22 μm pore filter. Incubation medium was withdrawn and fresh medium (90 μL) and MTT (10 μL) were added to the wells. Following the 2 h MTT incubation (at 37 °C), purple formazan crystals formed and 100 μL of DMSO was added. Absorbance was measured by a spectrophotometric plate redear at 570 and 690 nm. The absorbance formed in the control group was calculated to represent 100% cell viability [Cell viability (%) = (Abs sample/Abs control) × 100]. Three different experimental groups were formed for each cell line: control, DMSO (1 μL/mL), and 7-KSS (MCF-7 and HepG2 cells incubated with 30 μM 7-KSS for 24 h).

### 4.4. Immunofluorescent Staining

MCF-7 and HepG2 cells were transferred to chamber slides (100,000 cells/well) (Merck Millipore, Cork, Ireland). The chamber slides were incubated at 37 °C, 5% CO_2_ incubator overnight for cells to adhere. The culture medium was replaced with treatment medium when cells reached 70% confluency and incubation was carried out for 24 h. The medium was withdrawn and washed twice (0.01 M cold PBS). Then, 250 μL of freshly prepared 4% paraformaldehyde was added, and cells were fixed at room temperature for 10 min. The fixation solution was aspirated, and cells were washed 2 times with PBS buffer. For the permeabilization process, 0.2% Triton X-100 (Sigma-Aldrich, St. Louis, MO, USA) was prepared in 300 μL of PBS and was added and incubated for 30 min at room temperature. Cells were washed with cold PBS 5 times and 5% normal goat serum (NGS; Vector Laboratories, Burlingame, CA, USA) was added for blocking. The blocking solution was withdrawn, and cells were treated with phospho-p44/42 ERK1/2 (1:200 dilution, Cat.#9101, Cell Signaling Tech., Massachusetts, USA), phospho- NF-κB p65 (1:200 dilution, Cat.#AF2006, Affinity Biosciences, Changzhou, Jiangsu, China), and PCNA (1:200 dilution, Cat.#bs-0754R, Bioss Antibodies Inc., Woburn, MA, USA) antibodies at 200 μL per well overnight at 4 °C without washing. The next day, the chamber slides were washed 5 times at room temperature with PBS and incubated at room temperature for 45 min with Alexa Fluor-488 conjugated goat anti-rabbit (1:1000, Cat.#ab150077 Abcam, Cambridge, UK) secondary antibody at 200 μL per well in the dark room. After 3 washes with PBS, the chamber slide assembly was separated and a drop of DAPI (Vector Laboratories Inc., Burlingame, CA, USA) was dripped onto the slide for nucleus staining and closed with a coverslip without air bubbles. Slides were evaluated via a fluorescence microscope (Olympus IX81, Tokyo, Japan) at 20× magnification. Alexa Flour was imaged at 488 nm excitation and 505–525 nm emission, while DAPI was imaged at 350 nm excitation and 440–460 nm emission wavelengths. Fluorescence intensity in HepG2 and MCF-7 cells was determined by NIH ImageJ 1.53e software. Validated total cell fluorescence (CTCF) for single cell in each group was determined by the formula: CTCF = Integrated Density − (Area of the selected cell × Background mean fluorescence).

### 4.5. Enzyme-Linked Immunosorbent Assay

Proliferating cell nuclear antigen (PCNA) measurements were performed with a non-competitive sandwich ELISA kit (ELK Biotechnology; Denver, CO, USA, Cat.#ELK5141). MCF-7 and HepG2 cells were dosed according to the study groups and the collected cells (10^7^ cells/mL) were suspended in PBS and subjected to ultrasonication followed by centrifugation at 2–8 °C for 10 min at 1500× *g*. PCNA measurements were performed in supernatants following manufacturer’s instructions. The concentration of PCNA in the samples was determined at 450 nm via a PCNA standard curve and given as ng/mg protein.

Phospho-ERK analyses were performed with a non-competitive sandwich ELISA kit (ELK Biotechnology; Denver, CO, USA, Cat.#ELK8541). MCF-7 and HepG2 cells were dosed according to the study groups and the collected cells (10^7^ cells/mL) were suspended in PBS and subjected to ultrasonication followed by centrifugation at 2–8 °C for 10 min at 1500× *g*. Phospho-ERK measurements were performed in supernatants following manufacturer’s instructions. The concentration of p-ERK in the samples was determined at 450 nm via a p-ERK standard curve and reported as pg/mg protein.

Phospho-NF-κB p65 (ser536) measurements were performed with a non-competitive sandwich ELISA kit (BT Lab, Bioassay Technology Laboratory; Shanghai, China, Cat.#E4753Hu) according to manufacturer’s instructions. MCF-7 and HepG2 cells were dosed according to the study groups and the collected cells (10^6^ cells/mL) were suspended in PBS and subjected to ultrasonication followed by centrifugation at 2–8 °C for 20 min at 1500× g. The concentration of p-NF-κB p65 (ser536) in the samples was determined at 450 nm via a standard curve and reported as pg/mg protein.

### 4.6. Western Blot Analysis

Western blot analysis was performed as previously described [48]. 2-mercaptoethanol was added to Laemmli sample buffer (Cat.# 161-0737, BioRad Laboratories Inc., Hercules, CA, USA) prior to mixing with cell lysates obtained from treatment groups. Proteins were denatured at 100 °C and a 30 µL sample (30 µg protein) was separated on 12% Mini-protean TGX precast electrophoresis gels (Cat.#4561043, BioRad Laboratories Inc., USA). Trans-Blot SD Semi-Dry Transfer Cell (BioRad Laboratories Inc., USA) was used to transfer proteins from gels and immobilize them on nitrocellulose membranes, which were then immersed in 10 mL of blocking buffer [2% bovine serum albumin (BSA) in PBS] at room temperature for 5 min. Primary antibodies were diluted in blocking buffer and applied for 1 hour at room temperature. Primary antibodies were phospho-44/42 ERK1/2 (1:1000 dilution, Cat.#9101, Cell Signaling Tech., Danvers, MA, USA), phospho-NF-κB p65 (Ser536) (1:500 dilution, Cat.#AF2006, Affinity Biosciences, Jiangsu, China), NF-κB p65 (1:2000 dilution, Cat.#ab16502, Abcam Limited, Cambridge, UK), ERK1/2 (1:2000 dilution, Cat.#AF0155, Affinity Biosciences, Changzhou, Jiangsu, China), and beta Actin (1:1000 dilution, Cat.#ab8227, Abcam Limited, Cambridge, UK). Blots were washed in 10 mL Tris Buffered Saline (TBS) with 0.05% Tween 20 for 5 min a total of five times. Secondary antibody, Goat Anti-Rabbit IgG HRP-conjugate (1:10,000 dilution, Cat.# 632131, Sigma-Aldrich, St. Louis, MO, USA) was applied for 1 hour at room temperature and blots were washed in 10 mL TBS with 0.05% Tween 20 for 5 min a total of five times. Immunoreactive bands were visualized by ECL reagent (Amersham Pharmacia Biotech, Buckinghamshire, UK). All Western blots were quantified by image analysis using NIH ImageJ 1.53e software.

### 4.7. Determination of Apoptotic Cells

One-Step TUNEL Test Kit (Elabscience, Cat#E-CK-A320, Houston, TX, USA) was used to detect apoptotic cells. Terminal Deoxynucleotidyl Transferase (TdT) can catalyze the addition of fluorescein labeled dUTP to the exposed 3′-OH ends of the broken DNA. All reagents and samples were prepared in accordance with the test kit procedure. MCF-7 and HepG2 cells were transferred to chamber slides at approximately 100,000 cells per well. The culture medium was replaced with treatment medium when cells reached 70% confluency and incubation was carried out for 24 h. Positive control group was treated with DNase I, cutting off DNA to produce exposed 3′-OH ends. A total of 100 μL DNase I solution (200 U/mL) was applied, and cells were incubated at 37 °C for 30 min. The slides were washed with PBS 3 times for 5 min each time. For negative controls, cells were incubated with DNase I buffer at room temperature for 5 min and washed with PBS 3 times for 5 min each time. After the experimental groups completed the penetration step, 100 μL of TdT balancing buffer was added and slides were incubated at 37 °C for 25 min. At the end of the period, 50 μL of labeling working solution was added to each slide and incubated at 37 °C for 60 min in a humidified environment. The negative control labeling working solution did not contain TdT enzyme. At the end of the incubation period, the slides were washed with PBS 3 times for 5 min each time. The chambers were separated, wiped with a napkin, a drop of DAPI was dripped onto the slides, and a clean coverslip was sealed without air bubbles. The fluorescence intensity of the slides was then visualized under a fully automated Olympus BX61 microscope and analyzed using Image J software (NIH).

The apoptotic effects of 7-KSS on liver cancer and breast cancer cells were also evaluated with FITC-conjugated annexin-V and PI apoptosis kit (Elabscience: #E-CK-A211, Elabscience, Houston, TX, USA). Cell groups were established as previously described. At the end of the applications, the media were discarded. After the washing process, trypsinization was applied to remove the cells on the flask surface. To remove the cells from the trypsin-containing solution, the supernatant was discarded after centrifugation at 125× *g* for 5 min at +4 °C. Cells were suspended with PBS and 1 × 10^6^ cells were transferred to the flow cytometry tube. Centrifugation was carried out at 125× *g* for 5 min and the supernatant was removed. The cells were suspended with 500 µL annexin-V binding buffer. In total, 5 µL FITC-labeled annexin-V and 5 µL PI reagents were added to the cells and incubated for 20 min at room temperature. At the end of the incubation, fluorescent conjugated cells were quickly analyzed on a flow cytometry device (FACS Canto II, BD Bioscience, San Jose, CA, USA) at appropriate device settings. BD FACS Diva 6.1.3 software (BD Biosciences) was used for analysis.

### 4.8. Mass Spectrometric Sphingolipid Measurements

One ml of HepG2 and MCF-7 cell lysates (10 mg protein/mL) was prepared by adding 2 μL of 5 µg/mL IS stock solution. After the tubes were vortexed, chloroform: methanol (1:2, *v*/*v*) was added, sonicated for 30 s and vortexed for 5 min with the addition of 100 μL of distilled water. The resulting mixture was kept at room temperature for 30 min and after incubation, it was centrifuged for 5 min at 2000× *g* to obtain supernatants. A total of 125 μL of chloroform and 125 μL of distilled water was added to the supernatants, vortexed, and kept at room temperature for 30 min. After incubation, approximately 500 μL of the top-organic layer was transferred to a new glass tube and the volatilization process was carried out under a constant flow of nitrogen. The dried residues were dissolved in methanol:formic acid (100 µL; 99.9:0.1) and samples were transferred to insert vials and made ready for LC-MS/MS measurements. LC-MS/MS measurements were carried out as previously described [49].

### 4.9. Protein Measurements

Protein concentrations were measured at 595 nm using a modified Bradford assay with Pierce Bradford Plus Protein Assay Reagent (Thermo Fisher Scientific Cat.# 23238, Thermo Fisher, Waltham, MA, USA) and bovine serum albumin as the standard.

### 4.10. Statistical Analysis

The data were analyzed using the GraphPad Prism 9.00 and SigmaPlot 15 for Windows statistical program (Systat Software, Inc., St Palo Alto, CA, USA) A *p* value < 0.05 was accepted as statistically significant. The figure and table legends describe the statistical analysis for each measurement in detail. A normality test was performed prior to comparing the groups via the statistical analysis. When data were not normally distributed, we performed a nonparametric test.

## Figures and Tables

**Table 1:** Sphingolipid levels in cancer cells. All values mean ± SD. SM, sphingomyelin; S1P, sphingosine-1-phosphate; 7-KSS, 7-Ketositosterol. Statistical analysis was carried out by One-Way Analysis of Variance and all pairwise multiple comparison procedures were performed by the Tukey test. *, *p* < 0,001 vs. Control and DMSO groups.

	CONTROL	DMSO	7-KSS
**Sphingolipids (ng/mg protein)**			
**16:0 SM (d18:1/16:0)**			
MCF-7	159.83 ± 17.60	138.13 ± 21.36	143.80 ± 3.02
HepG2	154.40 ± 32.80	155.26 ± 27.77	178.63 ± 16.61
**18:0 SM (d18:1/18:0)**			
MCF-7	81.89 ± 8.14	84.93 ± 0.58	87.80 ± 9.12
HepG2	84.35 ± 6.49	89.53 ± 3.37	82.79 ± 5.70
**24:0 SM (d18:1/24:0)**			
MCF-7	34.51 ± 2.21	40.57 ± 7.38	39.94 ± 9.20
HepG2	40.38 ± 6.35	35.61 ± 5.96	39.22 ± 6.12
**C16 Ceramide (d18:1/16:0)**			
MCF-7	65.47 ± 7.88	65.89 ± 10.02	67.65 ± 2.76
HepG2	66.52 ± 8.58	66.20 ± 10.48	64.92 ± 8.75
**C18 Ceramide (d18:1/18:0)**			
MCF-7	7.40 ± 0.30	6.87 ± 0.71	37.20 ± 1.86 *
HepG2	6.26 ± 0.29	6.22 ± 0.16	34.89 ± 1.72 *
**C20 Ceramide (d18:1/20:0)**			
MCF-7	7.95 ± 0.27	7.60 ± 0.22	45.70 ± 2.67 *
HepG2	6.37 ± 0.18	6.54 ± 0.34	31.56 ± 0.52 *
**C22 Ceramide (d18:1/22:0)**			
MCF-7	24.11 ± 3.10	23.84 ± 4.22	64.63 ± 3.48 *
HepG2	23.97 ± 3.30	24.08 ± 3.04	66.50 ± 2.79 *
**C24 Ceramide (d18:1/24:0)**			
MCF-7	44.02 ± 2.48	43.20 ± 0.62	121.34 ± 13.16 *
HepG2	42.47 ± 5.03	35.30 ± 3.69	101.38 ± 1.77 *
**S1P**			
MCF-7	5.36 ± 0.27	5.00 ± 0.52	1.79 ± 0.08 *
HepG2	8.39 ± 0.27	7.92 ± 0.47	2.58 ± 0.15 *

## Data Availability

Data obtained and analyzed in this work are available from the corresponding author on reasonable request.

## Abbreviations

7-KSS: 7-ketositosterol; AP1, activated protein 1; CERs, ceramides; CTCF, validated total cell fluorescence; ERKs, extracellular signal-regulated kinases; MAPKs, mitogen-activated protein kinases; NF-κB, nuclear factor kappa B; PCNA, proliferating cell nuclear antigen; PI, propidium iodide; POPs, phytosterol oxidation products; S1P, sphingosine-1-phosphate; SMs, sphingomyelins; TLR4, toll-like receptor 4; UV, ultraviolet.
